# Primary Hydatid Cyst of the Adrenal Gland: A Case Report and Review of the Literature

**Published:** 2011-05-01

**Authors:** B Geramizadeh, M Maghbou, B Ziyaian

**Affiliations:** 1 Department of Pathology, Transplant Research Center, Shiraz University of Medical Sciences, Shiraz, Iran; 2 Department of Surgery, Shiraz University of Medical Sciences, Shiraz, Iran

**Keywords:** Adrenal gland, Hydatid cyst, Iran

## Abstract

Hydatid cyst of adrenal gland is rare and usually incidentally found as a part of disseminated disease. Herein we report a rare case of primary adrenal hydatid cyst who presented with unusual symptom of arterial hypertension from an endemic country.

## Introduction

Hydatid cyst, caused by Echinococcus granulosus larva, most commonly can be found in countries of Middle East, Eastern Europe, Africa, Latin America and China.[1] Echinoccocus seems to be endemic in Iran.[2] Most common locations are liver (59-75%) and lung (27%). Hydatid cyst of adrenal gland is very rare comprising 0.5% of all cases.[3] Herein we report a patient with an Echinococcus cyst of left adrenal who presented with hypertension and operated with the radiologic and clinical impression of pheochromocytoma.

## Case Report

**Fig. 1:**
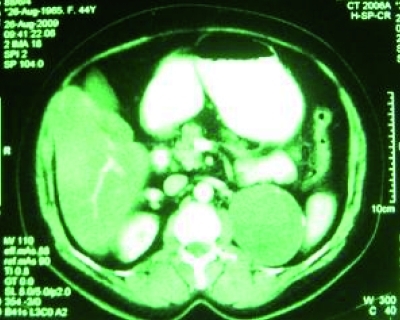
CT scan shows hypoechoic mass in the upper pole of left kidney

A 49-year-old diabetic woman referred with left flank pain. Her physical examination was unremarkable, except for high blood pressure (160/90 mmHg). Complete blood count and electrolytes were normal. The biochemistry analysis showed high triglyceride (260 mg/dl) and fasting blood sugar (180 mg/dl). Other laboratory results were normal, including renin, aldosterone and 24-hour urinary VMA and metanephrine. Abdominal sonography demonstrated an 82 mm hypoechoic mass, containing internal cystic component adjacent to superior pole of left kidney. CT scan of abdomen showed an 8.2x7.5x6 cm hypoattenuated cystic mass with calcified wall in the left adrenal gland (Figure 1).

Because of the newly diagnosed arterial hypertension, she was operated with the possible impression of pheochromocytoma. During operation a cystic mass with solid components was detected in the left adrenal measuring 8x7x6 cm, which had pushed the kidney downward. The cystic mass was successfully removed with intact wall by adrenalectomy and sent for pathologic study. Gross sectioning of the specimen showed adrenal gland with some cystic structures. These cysts had whitish friable wall, grossly in favor of hydatid cyst. Microscopic examination confirmed the gross findings and multiple cystic structures with some scolices and laminated membrane were seen. After operation, all blood pressures were about 130/80 mmHg and postoperative period was uneventful. After 2 months, she was doing well and symptom-free.

## Discussion

Hydatid cysts are most commonly found in the liver and lung, while they can occur in other organs including myocardium, brain, eye, spleen, and kidney.[4] The adrenal hydatid cyst is extremely rare and less than 20 cases have been described till 2007.[2]

As a whole, parasitic cysts of adrenal gland are very rare and accounts for about 7% of the cysts in the adrenal gland.[5] Most of the hydatid cysts arising from adrenal are part of disseminated disease and primary adrenal hydatid cyst is very unusual.[5] Most of the adrenal hydatid cysts are asymptomatic and incidentally found by imaging or during surgery for other abdominal pathologies.[6] However common presenting symptoms in the previously reported cases have been vague abdominal and flank pain, gastrointestinal complaints or palpable mass.[5]

Adrenal hydatid cyst presenting with arterial hypertension has rarely been reported.[6][7][8][9][10] Immunodiagnostic tests are helpful with a diagnostic sensitivity of 90%, but radiology has an important role in preoperative diagnosis of adrenal masses,[6] and ultrasonography can be the first choice (sensitivity 93-98%), because it is easy to perform and inexpensive. Presence of calcification and septation in an adrenal cyst in CT scan are in favor of parasitic cyst.[5]

In our patient, despite of the presence of calcification in imaging studies, newly diagnosed arterial hypertension has miss-led everybody to the diagnosis of pheochromocytoma. Most authors recommend adrenalectomy for the treatment of hydatid cysts, but a few reports have proposed simple cystectomy.[5] Our case has been treated with adrenalectomy and the cyst was excised intactly. All blood pressures after surgery were normal, which confirms the link between the cyst and arterial hypertension. In our patient after the pathologic diagnosis of adrenal hydatid cyst, all the imaging studies were failed to show any other site of involvement. Therefore, the patient was labeled as primary adrenal hydatid cyst who presented with arterial hypertension.

As a conclusion, in endemic regions such as Iran, the hydatidic etiology of adrenal cysts should be considered and no attempt to preoperative puncture or any other manipulation of the lesion should be done.
